# Evaluating patient diversity in early phase clinical trials in Australia through a prospective multicenter nonrandomized cohort study

**DOI:** 10.1093/jncics/pkaf035

**Published:** 2025-03-27

**Authors:** Udit Nindra, Joanne Tang, Jun Hee Hong, Martin Hong, Christina Teng, Joe Wei, Andrew Killen, Adam Cooper, Kate Wilkinson, Weng Ng, Charlotte Lemech, Wei Chua, Abhijit Pal

**Affiliations:** Department of Medicine, Western Sydney University, Campbelltown, Australia; Department of Medical Oncology, Wollongong Hospital, Wollongong, Australia; Department of Medical Oncology, Liverpool Hospital, Liverpool, Australia; Department of Medical Oncology, Liverpool Hospital, Liverpool, Australia; Department of Medical Oncology, Liverpool Hospital, Liverpool, Australia; Department of Medical Oncology, Liverpool Hospital, Liverpool, Australia; Scientia Clinical Research, Randwick, Australia; Department of Medical Oncology, Prince of Wales Hospital, Randwick, Australia; Scientia Clinical Research, Randwick, Australia; Department of Medical Oncology, Prince of Wales Hospital, Randwick, Australia; Scientia Clinical Research, Randwick, Australia; Department of Medical Oncology, Liverpool Hospital, Liverpool, Australia; Department of Medical Oncology, Liverpool Hospital, Liverpool, Australia; Department of Medicine, Western Sydney University, Campbelltown, Australia; Department of Medical Oncology, Liverpool Hospital, Liverpool, Australia; Scientia Clinical Research, Randwick, Australia; Department of Medical Oncology, Prince of Wales Hospital, Randwick, Australia; Department of Medicine, Western Sydney University, Campbelltown, Australia; Department of Medical Oncology, Liverpool Hospital, Liverpool, Australia; Department of Medical Oncology, Liverpool Hospital, Liverpool, Australia

## Abstract

**Background:**

Early phase clinical trials continue to have difficulty with enrolling real-world populations with many minorities being underrepresented. Reasons for this include patient or clinician perception as well as cultural, linguistic, or social barriers. In Australia, there is currently no prospective data in the early phase clinical trial space regarding recruitment of priority populations.

**Methods:**

Patient Diversity in Early Phase Clinical Trials was a multicenter, prospective, cohort study involving 2 major early phase clinical trial centers in Sydney, Australia. All participants who were consented to an early phase clinical trial between August 2023 and August 2024 were enrolled. Participants completed a baseline demographic survey, which included cultural and linguistic status, sexual orientation, socioeconomic status, and regional diversity.

**Results:**

A total of 114 participants were recruited. Median age was 63 years (range = 25-83 years) with predominance for female participants (52%). No participant reported a nonbinary gender. All participants reported their sexuality as heterosexual, with no LGBTQIA+ participants recruited. A total of 34 (30%) participants were identified as culturally diverse, while 28 (25%) were linguistically diverse. One patient identified as Indigenous Australian. Of the participants, 26% were born overseas, with 44% having at least 1 parent born overseas. The majority were living in households with family members, with 8% of participants living alone.

**Conclusion:**

Patient Diversity in Early Phase Clinical Trials is the first prospective study that provides granular description of social, cultural, linguistic, economic, and sexual diversity among early phase clinical trial participants. Certain subgroups are underrepresented, including those with sexual diversity, gender diversity, and Indigenous backgrounds. Ongoing efforts to monitor and promote inclusion of diverse populations in clinical trials are vital.

## Introduction

Early phase clinical trials, otherwise known as phase I studies, are designed to investigate the dose, pharmacokinetic, and safety profile of new therapeutic agents. Their primary goal is often to determine dose-limiting toxicity and expectant side effects. In patients with advanced cancer, these trials represent hope of providing additional tumor control once standard of care options have been exhausted, which needs to be balanced against unforeseen risks and uncertain clinical benefit. Over the past few decades, as clinicians’ ability to recommend specific studies with better scientific merit to patients has enhanced, as well as scientific advances and improvements in drug development, the overall clinical benefit rate of early phase clinical trials in oncology has also improved.^1^

Patients on cancer clinical trials are known to poorly reflect clinical and social characteristics of real-world populations. Because of the unknown safety profile of new drugs, stringent eligibility requirements exclude many cancer patients who have impaired physiology due to the nature or complications of their disease. Furthermore, many minority populations are not represented in clinical trials because of a number of factors including patient or clinician perception as well as cultural, linguistic, or social barriers.^2^ This remains an issue for early phase clinical trials as well as later phase trials. Clinicians have aimed to investigate which interventions may be appropriate to help improve such disparities, but gaps in enrollment remain persistent.^3^ To date, solutions to bridge the gap have focused on clinician education regarding appropriate cultural communication techniques,^4^ investing in cancer trial coordinators who are linguistically and culturally matched,^3^ and providing visual cues of inclusivity where possible to promote diverse recruitment.^5^

To complicate the issue, there has been poor documentation of participant diversity characteristics in cancer clinical trials.^6^ Many studies in the United States have inadequately reported participant ethnicity data, with a 20-year systematic review comprising more than 20 000 clinical trials and over 4 700 000 participants reporting that race and ethnicity data were recorded in less than 50% of cases.^7^ White participants remained the most commonly recruited population at 80%, with Indigenous patients recruited at a rate of less than 1%.^7^ In Australia, there also remains a paucity of data to determine if there has been any shift in recruitment diversity over time, with many studies looking at retrospective cohorts. Efforts to promote equal health outcomes in priority populations such as those with sexual diversity and those with minority and Indigenous backgrounds have been made worldwide, but the impact of these interventions is not well established.^8^ In Australia, there is currently no prospective data in the early phase cancer clinical trials space regarding these priority populations.

Intersectionality studies have shown that a person’s identity can expose them to overlapping forms of discrimination and marginalization and is becoming central to cancer equity discussions.^9^ Consequently, it remains crucial that studies prospectively capture diversity data to assess barriers to clinical trial enrollment. Current data capture is unregulated and often the bare minimum—for example, country of birth and language preference are the only data points traditionally captured in Australian cancer clinical trials and, even if recorded, are often not reported in the final manuscript. These 2 data points are insufficient to understand the way multiple factors intersect to produce poor cancer outcomes such as missing out on clinical trial participation. Comprehensive data capture of the various dimensions of vulnerability is needed to better understand how to target equity interventions. To move the dial forward for this important equity issue, the Patient Diversity in Early Phase Clinical Trials (PEARLER) was designed.

## Methods

The PEARLER study was a multicenter, prospective, cohort study involving 2 major early phase clinical trial centers in Sydney, Australia. All participants who were consented to an early phase clinical trial at either center were invited to consent between August 2023 and August 2024. There were 3 key aims of this study. The first was to prospectively assess social, cultural, linguistic, economic, and sexual diversity among participants enrolling into an early phase clinical trial and to compare these findings with the diversity of the greater Australian population. In addition, PEARLER was designed to capture patient-reported, health-related, quality-of-life outcomes of participants enrolling in early phase clinical trials. Lastly, it also aimed to capture the overall progress made in clinical outcomes for participants enrolling in early phase clinical trials and compare these with local retrospective cohorts. This analysis focuses on the first aim of the study.

All participants completed a baseline demographic survey on cycle 1 day 1 (Figure S1). Demographics collected included patient cultural and linguistic status, sexual orientation, socioeconomic status (SES), and regional diversity. We also collected the patient’s self-reported level of English proficiency based on a 5-tier scale from “A” denoting an English-only speaker to “E” denoting use of another language with limited to no English proficiency (Figure S1). This was based on the Australian Bureau of Statistics Proficiency in Spoken English assessment.^10^

The patient’s parental country of birth to further clarify cultural diversity status was recorded. Patients were defined as culturally diverse if they, or at least 1 of their parents, were born in a country overseas where English was not the predominant language. Distance traveled to the early phase clinical trial center using the patient residential address was noted. In cases where the exact residential address was not available, the patient’s postcode was used to estimate the distance traveled.

SES was quantified by the Index of Relative Socioeconomic Advantage and Disadvantage (IRSAD) scores developed by the Australian Bureau of Statistics. These scores were calculated from Socioeconomic Indexes for Areas data using the patient’s residential postcode at the time of enrollment onto the early phase clinical trial. The IRSAD score quantifies the economic or social advantage and disadvantage experienced by patients based on their region of residence. A score of 1000 represents the median for Australia. A score below 1000 indicates a patient resides in an area with greater disadvantage compared with the median of Australia. Every 100 points below 1000 represents 1 standard deviation of difference in the disadvantage index. Participants were classified into the ISRAD quintile in which their primary residence was on a basis of 1 (most advantaged) through 5 (most disadvantaged).

Participants completed health-related quality-of-life assessments using the European Organisation for Research and Treatment of Cancer Quality of Life Questionnaire Core 30 on day 1 of cycles 1 through 6. The project was approved by the Sydney South West Local Health District human research ethics committee (2023/ETH00786).

## Results

A total of 125 participants were enrolled into early phase clinical trials across the 2 sites, of which 114 were recruited to the PEARLER study over the 12-month period. The median age of the participants was 63 years (range = 25-83 years) with a slight predominance for female participants (52%). No participant reported their gender as nonbinary. All participants reported their sexuality as heterosexual, with no LGBTQIA+ participants identified.

The majority of participants had a baseline Eastern Cooperative Oncology Group score of 0 (n = 76, 67%), and most were nonsmokers (57%). The most common cancer types recruited to early phase clinical trials were gynecological (21%) and lung (21%). Among the 114 participants, 34 (30%) participants were identified as culturally diverse, while 28 (25%) were linguistically diverse. Despite this high level of linguistic diversity, English was the preferred language in the vast majority (87%) of participants. Of the 14 participants whose preferred language was one other than English, only 6 (43%) required the use of an interpreter during the trial consent process. The most common preferred language other than English was Mandarin (4 participants). More than one-quarter (26%) of participants were born overseas, with almost half (44%) having at least 1 parent born overseas. The vast majority of participants were living in households with family members, with only 8% participants reporting that they lived alone. Almost half of participants enrolled into early phase clinical trials in PEARLER were living in areas above the median IRSAD score for socioeconomic advantage and disadvantage, with 23 (20%) living in the lowest SES quintile.

The median distance between the participants’ primary residence and the early phase clinical trial center was 31 km. Of the participants, 29% lived within 10 km of the trial site location, and almost 40% had their primary residence greater than or equal to 50 km away from the trial site. There were no differences in culturally and linguistically diverse rates between those who lived closer (<30 km) vs further away from their early phase clinical trial location. In terms of the clinical studies, the majority (70%) was in the dose escalation phase. Targeted therapy investigational products were used in 71% of early phase clinical trials, while the remainder focused on immuno-oncology protocols. A full breakdown of the participant demographics can be found in Table 1.

**Table 1.** Participant demographics

Demographic	No. (%)
Total participants	114
Age, median (range), y	63 (25-83)
Self-reported gender	
Female	59 (52)
Male	55 (48)
Other	0 (0)
Self-reported sexuality	
Heterosexual	114 (100)
LGBTQIA+	0 (0)
Eastern Cooperative Oncology Group Performance Score	
0	76 (67)
1	38 (33)
Smoking status	
Never smoked	65 (57)
Current or ex-smoker	49 (43)
Cancer type	
Gynecological	24 (21)
Lung	24 (21)
Upper gastrointestinal	14 (12)
Head and neck	12 (11)
Colorectal	11 (10)
Brain	7 (6)
Skin	6 (5)
Breast	3 (3)
Other	21 (18)
Culturally and linguistically diverse status	
Culturally diverse	34 (30)
Linguistically diverse	28 (25)
Indigenous Australian	1 (1)
Country of birth	
Australia	8 (74)
Overseas	30 (26)
≥1 parent born overseas	50 (44)
Preferred language	
English	100 (88)
Other	14 (12)
Socioeconomic status	
Index of Social Advantage and Disadvantage 4-5	57 (50)
Index of Social Advantage and Disadvantage ≤3	57 (50)
Living status	
Living with family	105 (92)
Living alone	9 (8)
Clinical trial phase	
Dose escalation	80 (70)
Dose expansion	34 (30)
Type of investigational product	
Targeted therapy	71 (62)
Immuno-oncology	43 (38)

## Discussion

Our study is the first in Australia to prospectively assess social, cultural, linguistic, economic, and sexual diversity among participants enrolling into early phase clinical trials. Australia is regarded as one of the most multicultural countries in the world, with almost 30% of the population born overseas and more than 25% speaking a language other than English.^11^^,^^12^ As cancer care becomes increasingly complex and with treatment algorithms rapidly changing, there is an increasing requirement for practitioners to be aware of how to conduct discussions regarding experimental and standard therapy with diverse populations. This is even more important in early phase clinical trials where risks of therapy are often not as clearly defined, and despite improvements over time in clinical outcomes of those partaking in early phase clinical trials, the benefits are not guaranteed.^13^

Culturally, diversity is limited not only to the country of birth of an individual but also of their parents as this determines their ancestral roots. Although many studies have looked at country of birth to define culturally diverse status, in our prospective study patients were defined as culturally diverse if they, or at least 1 of their parents, were born in a country overseas where English was not the predominant language. We hypothesize that the longer an individual is in a country (such as those newly arrived first-generation migrants compared with second-generation migrants), the less susceptible they are to poorer health-care outcomes. Using this definition, 34 (30%) participants were defined as culturally diverse, with 30 (26%) born overseas. Currently, there is no prospective data assessing culturally and linguistically diverse recruitment in early phase clinical trials in Australia, with previous retrospective studies across Europe and the United States reporting up to 40% culturally and linguistically diverse populations in some datasets.^2^^,^  ^14^^,^^15^ In comparison, the PEARLER prospective data is in line with the wider Australian population in which currently 30% are born overseas. This potentially suggests that ongoing efforts to improve culturally and linguistically diverse patient recruitment to clinical trials have been beneficial.

Cultural diversity may not be the primary limiting factor to early phase clinical trial participation. An Australian study analyzing more than 19 000 participants noted that among culturally and linguistically diverse participants, presence of limited English proficiency was the key driver of poor trial recruitment.^16^ The American Society of Clinical Oncology Joint Research Statement on culturally and linguistically diverse recruitment in clinical trials notes that there are multiple barriers to recruitment that can be at the patient level as well as among clinicians themselves.^2^ However, the medical community in Australia is also becoming increasingly culturally diverse;^17^ perhaps this is resulting in a cultural attitude shift and, thereby, improvement in culturally and linguistically diverse participant recruitment. This has been noted in previous studies, where “race matching,” defined as alignment of the race of the patient and the researcher, has been suggested to enhance clinical trial participation.^4^ Culturally and linguistically diverse status and a lower SES are 2 factors that are often connected, and socioeconomic constraints have been cited by many researchers as barriers to recruitment of culturally and linguistically diverse participants in clinical trials.^4^^,^  ^18^^,^^19^ Hence, recruitment from culturally and linguistically diverse populations requires ongoing efforts to understand the intersectionality at play that limits trial participation, with a focus on creating equitable systems that can support participants enrolling into cancer trials rather than a focus on efforts that extend beyond cultural-specific interventions.

Indigenous Australians are 39% more likely to die from cancer-related complications compared with non-Indigenous Australian counterparts.^9^ As early phase clinical trials often represent the last line of treatment for many patients, recruitment of Indigenous Australians is important for holistic diversity in cancer care and ensuring equitable cancer outcomes for all Australians. There is currently no data to document the rate of Indigenous Australian recruitment to early phase clinical trials in Australia. In Australia, 4% of the population identify as an Indigenous Australian. The PEARLER data identified only 1 (<1%) patient in this category, potentially reflecting further issues with diversity inclusion in early phase clinical trials. Previous studies from the United States also demonstrate low rates of Indigenous patient recruitment, with multiple barriers identified including lack of trust and fear of health services.^20^ The low levels of participation of Indigenous Australians in the PEARLER datasets is consistent with findings from other trial populations.^21^ Currently, there are no prospective studies in the early phase clinical trial space that attempt to identify specific barriers that limit Indigenous Australian recruitment, but further efforts with culturally appropriate educational materials, aboriginal liaison support involvement during the recruitment process, and clinician sensitivity and training around social and cultural aspects are potential pathways of improvement for the future.

Language barriers are considered to be one of the key challenges when discussing clinical trials with non-English-speaking participants and can lead to reduction in patient satisfaction in health care.^22^ Often, clinicians rely on interpreters to help bridge the gap, but there continue to remain difficulties with accessing timely provision of these services in Australia.^3^ In the PEARLER cohort, 28 (25%) participants were classified as linguistically diverse, which was relatively consistent with the wider Australian population.^10^ Of these, 14 (10%) participants stated that a language other than English was their preferred language, with 6 (43%) requiring the use of an interpreter during health-care interactions. There have been multiple interventions that have been considered to help limit the impact of language barrier on clinical trial participation. Recently, a survey of Australian oncologists was completed to determine solutions for this, with trial navigators and a generic cancer trial pamphlet available in multiple languages being judged as the top solutions to improve recruitment.^3^ Despite this, access to culturally diverse clinical trial navigators and translated clinical trial information remains limited. This is despite real-world evidence that providing linguistically diverse clinical trial information can improve access and subsequent recruitment to clinical trials.^23^

Participants identifying as LGBTQIA+ frequently experience discrimination and structural stigma, leading to poorer access and engagement in health-care and, consequently, access to early phase clinical trials. At least 3%-4% of Australians identify as nonheterosexual, and approximately 1% as gender diverse.^24^^,^^25^ Despite this, there has been no research on representation of LGBTQIA+ participants in cancer early phase clinical trials. In the PEARLER study, no participants identified their gender as nonbinary. All participants self-identified their sexual orientation as heterosexual; no participants listed their sexual orientation as “same sex” or “other.” This result could be a product of chance given the rates of gender diversity in Australia or potentially could be a product of unwillingness of participants to reveal their gender diversity. Previously, it has been demonstrated that members of the LGBTQIA+ may withhold such information or be health-care avoidant because of fear of discrimination.^26^ Strategies to ensure inclusivity of LGBTQIA+ participants in clinical trials include education of health-care workers to provide LGBTQIA+ friendly communication^27^ along with visual displays in clinical settings that promote gender-diverse inclusivity.^5^ Overall, early phase clinical trials offer a valuable opportunity in cancer management, and thus promotion of equitable recruitment of cancer participants including LGBTQIA+ participants into early phase clinical trials is warranted for all cancer trial centers in Australia. of 

Social isolation is associated with poorer cancer-related outcomes, with studies demonstrating participants who live alone have not only a higher incidence of cancer but also a higher excess cancer-specific mortality.^28^^,^^29^ Currently, no previous study to date has examined the incidence of social isolation in participants enrolling into early phase clinical trials. Through PEARLER, we determined that 9 (8%) participants nominated that they were living alone as compared with family. This is lower than the general Australian population where it is noted that more than a quarter of households are now occupied by only 1 person.^12^ Considering the demographic trend toward increasing single-person households in Australia over the past 50 years, prospective reporting of such disparities underscores the need for cancer centers to account for limited social supports when ensuring equitable trial recruitment.

The opportunity to participate in early phase clinical trials can also be a product of access to clinical trial sites. Many participants travel large distances for cancer treatment, and although this has previously been shown not to impact clinical trial outcomes,^30^ it can be associated with lower clinical trial enrollment.^4^ In PEARLER, the median distance traveled by participants from their primary residence to an early phase clinical trial site was 31 km. Traveling such distances for treatment can be associated with time toxicity,^31^ increasing financial burden, and culminate in a decision not to participate in early phase clinical trials. The impact of trial site location has been shown to be of relevance not only to SES disadvantaged populations but also to minority and priority populations.^32^ Hence, ongoing efforts to increase early phase clinical trials in locations with higher priority populations, as well as regional and rural locations, are needed. Additionally, further improvement in telehealth technologies and telehealth trials could limit the need for participants to travel significant distances for clinical trial visits. This has also led to increasing interest for decentralized clinical trials as a new paradigm of trial delivery to improve equity of access.^33^ This model appears to be an attractive option, and significant funding through the Australian government has been provided to create the National Teletrials Network to promote the safe implementation of these studies as well as ethical management of patients and their data. The Australian Gastrointestinal Trials Group is also running 6 current decentralized teletrials but none within the early phase clinical trial space. Challenges with governance and safety exist for patients in such situations, and ongoing efforts to provide frameworks for safe decentralized early phase clinical trials in Australia are needed.

As outlined by the American Society of Clinical Oncology policy statement on social determinants of health and cancer care, social determinants of health affect all aspects of the cancer care continuum, from screening through end of life and/or survivorship. As such, SES can also play a role in clinical trial recruitment. Time constraints, including those connected with job responsibilities, can also be preventive factors in study participation.^4^ In the PEARLER cohort, 23 (20%) participants resided in the lowest SES quintile, with a further 21 (18%) residing in the second most SES disadvantaged regions of Australia. This potentially highlights the inclusive nature of clinical trials in the Australian setting, especially in a cancer health-care system that is vastly publicly funded. Participants are not charged for their reviews with oncologists in public health services, and at neither site in our study were participants expected to pocket any expenses for trial-related activities. Given this recognition of the financial toxicities of health care, especially when considering the large distances many of our participants travel for early phase clinical trial inclusion, this SES diversity is a positive reflection of cancer clinical trials at the participating centers.

Equity of representation in early phase clinical trials is critically important so that toxicity and efficacy data are representative of patients in the real world who are ultimately going to receive these drugs if successful. The PEARLER study is the first prospective study of real-world data that investigates social, cultural, linguistic, economic, and sexual diversity among participants enrolling into early phase clinical trials. These data demonstrate that with ongoing efforts for improvement in inclusivity, there appears to be consistent recruitment of participants with diverse backgrounds that reflect the diversity of the Australian population. Despite this, certain subgroups continue to be underrepresented, such as those with sexual and gender diversity and minority Indigenous backgrounds. Although the PEARLER study is closed, there remain ongoing efforts to promote inclusion of diverse populations among clinical trials. Ongoing data collection regarding demographic trends is occurring across both sites included in the study with the aim to recruit a real-world population that can help guide clinicians and researchers to better manage cancer treatments and ensure equity of cancer outcomes.

## Supplementary Material

pkaf035_Supplementary_Data

## Data Availability

The datasets for this manuscript are not publicly available, but requests to access the datasets should be directed to Udit Nindra (udit.nindra@health.nsw.gov.au).
