# Supplementary material for: Evaluating patient diversity in early phase clinical trials in Australia through a prospective multicenter nonrandomized cohort study
Source: JNCI Cancer Spectr. 2025 Mar 27;9(2):pkaf035. doi: 10.1093/jncics/pkaf035 (PMC12020725; doi:10.1093/jncics/pkaf035)
Supplement: pkaf035_Supplementary_Data [file pkaf035_supplementary_data.zip › Supplementary Appendix.docx]

**Supplementary Figure 1**

1. Centre of recruitment
   - - Westmead
     - Scientia
     - St Vincent’s
     - Macquarie
     - Liverpool
2. Date of Birth:
3. Local MRN:
4. Age:
5. Trial Type:
   - - Immuno-oncology
     - Targeted Therapy
     - Other (specify):
6. Consent Date:
7. Trial commencement date (planned or known):
8. Gender:

- Male
- Female
- Non-binary (specify):
- Prefer not to say:

1. Country of Birth:
2. Country of Parent 1 Birth:
3. Country of Parent 2 Birth:
4. Sexual Orientation:
   - - Heterosexual
     - Same-sex
     - Other (specify)
     - Prefer not to say
5. Residential Post code:
6. Main language spoken at home:
7. Preferred language:
8. Proficiency in spoken English:
   - - Speaks English Only
     - Uses other language but speaks English very well
     - Uses other language but speaks English well
     - Uses other language but speaks English not well
     - Uses other language but speaks English not at all
9. Interpreter used during consent (Yes/No)
10. Indigenous status
    - - Yes/No
      - If yes, which language
11. Functional status
    - - ECOG 0
      - ECOG 1
      - ECOG 2
12. Smoking Status
    - - Yes, current
      - Yes, ex-smoker
      - Never smoked
13. Living condition
    - - Living with partner/extended family
      - Living alone
14. Stage of Phase 1 Trial
    - - Dose escalation
      - Dose expansion
15. Cycle Length:
16. Route of administration of Trial Medication:
    - - Oral
      - Intravenous
      - Other:
17. Biopsy required for trial entry (Yes/No)
18. Original Cancer Tumour Stream
    - - CNS
      - Lung
      - Colorectal
      - Skin
      - Genitourinary (Prostate, Renal, Bladder, Urothelial)
      - Gynaecological
      - Head and Neck
      - Breast
      - Unspecified (Cancer of Unknown Origin)
      - Other:
19. Date of Diagnosis
20. Stage of cancer at trial entry
21. Metastatic sites:
22. Number of metastatic sites
23. Number of previous lines of therapy
24. Baseline blood test results:
    - - Na
      - K
      - Albumin
      - LDH
      - Platelets
      - Neutrophils
      - Lymphocytes
      - eGFR
      - Creatinine
      - ALT
      - AST
      - GGT
      - ALP
      - Bilirubin
      - INR
      - APTT
